# Aspects of visual avatar appearance: self-representation, display type, and uncanny valley

**DOI:** 10.1007/s00371-021-02151-0

**Published:** 2021-06-17

**Authors:** Daniel Hepperle, Christian Felix Purps, Jonas Deuchler, Matthias Wölfel

**Affiliations:** 1 Faculty of Computer Science and Business Information Systems, Karlsruhe University of Applied Sciences, Karlsruhe, Germany; 2 Faculty of Business, Economics and Social Sciences, University of Hohenheim, Stuttgart, Germany

**Keywords:** Virtual reality, Avatar customization, Nonverbal communication, Head-mounted displays, Social networks

## Abstract

The visual representation of human-like entities in virtual worlds is becoming a very important aspect as virtual reality becomes more and more “social”. The visual representation of a character’s resemblance to a real person and the emotional response to it, as well as the expectations raised, have been a topic of discussion for several decades and have been debated by scientists from different disciplines. But as with any new technology, the findings may need to be reevaluated and adapted to new modalities. In this context, we make two contributions which may have implications for how avatars should be represented in social virtual reality applications. First, we determine how default and customized characters of current social virtual reality platforms appear in terms of human likeness, eeriness, and likability, and whether there is a clear resemblance to a given person. It can be concluded that the investigated platforms vary strongly in their representation of avatars. Common to all is that a clear resemblance does not exist. Second, we show that the uncanny valley effect is also present in head-mounted displays, but—compared to 2D monitors—even more pronounced.

## Introduction

At the time of Facebook Inc. acquisition of Oculus$^{\mathrm{TM}}$ in March 2014, many thought it would revolutionize the way people interact with each other in social media, gaming, and education. While there may be a high potential for all of these disciplines, actual usage is progressing rather slowly.^1^ Recent global challenges like global warming and biohazards require many people to stay at home and work in almost complete social isolation.^2^ Telepresence, which can reduce the feeling of being socially isolated, is therefore in demand as never before. To date, however, social virtual reality platforms have not yet attracted a comparably large number of users as other social media platforms such as Facebook, Instagram, Snapchat, Twitter, Likee, or TikTok. The reason could be due to various challenges in *virtual reality* (VR) that need to be overcome, such as additional costs, limitations in movement, cybersickness, comfort, and self-expression as well as self-representation.

While video conferencing may still be the first choice for most people to overcome physical distance, alternative technologies such as VR offer opportunities to replace “real-life” encounters more comprehensively and intuitively by allowing people to interact with each other in a shared 3-dimensional space. This shared space has a high potential to lead to higher (perceived) immersion, presence, and connection to others than comparable content being perceived using a regular screen. The main difference between the two modalities is the way participants represent themselves. While in a video chat session one is represented by a camera image and ultimately the background is blurred or altered and sometimes filters are applied to facial features such as red cheeks or the like, in *social virtual reality* (SVR) one has to find a suitable representation of oneself using a dedicated 3D model.

To create a respective avatar three different approaches, requiring more or less knowledge and hardware, are possible:

- using an avatar customizing toolbox which is usually provided within the platform,
- building your own model by using either tools to customize humans such as MakeHuman, Poser, DAZ Studio or 3D modeling software such as Blender, Cinema 4D, Maya or
- use multi-camera rigs that only a small number of potential VR users have access to.

Other approaches such as using artificial intelligence to recreate a 3D model of a person from 2D images or videos are promising but have not yet been rolled out to the general public because most of the time, it still requires post-processing via 3D modeling tools for rigging and texturing [1] or are not close in resemblance.

With the increased complexity of how someone is represented in a 3D space, one might ask how current SVR platforms deal with this situation, especially when thinking about self-identification, resemblance, or the *uncanny valley effect* (UV), which, if not being taken into consideration, can have an unwanted negative impact on how someone’s representation is perceived by other participants. More on the uncanny valley effect has been investigated in our previous study[2].

**Fig. 1:**
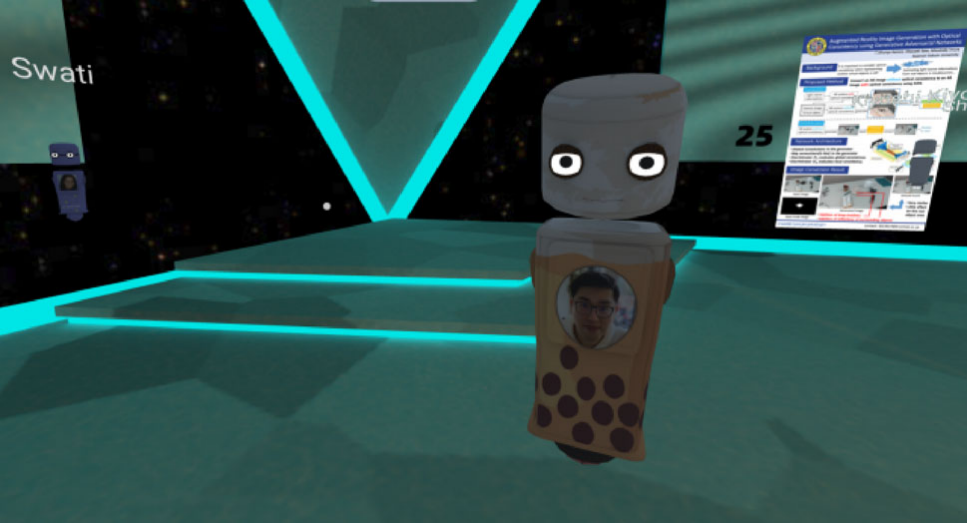
Screenshot showing one participant of IEEE VR 2020 conference with an image of his face mapped on the body of the 3D avatar. Courtesy of Eva Wolfangel. Used with permission

Due to the shutdown caused by the SARS-CoV-2 virus, scientific conferences are searching for solutions that can take place in the virtual instead of the real. In 2020 the renowned VR conference IEEE VR was set up as a SVR conference using Mozilla Hubs (Hubs) where participants could join using a standard desktop environment or join using a VR headset. While Hubs offers templates to generate your very own avatar, there are also predefined avatars that can be customized to a certain degree. Even though we cope with a very professional user group highly skilled in computer graphics many avatars were quite basic without providing any form of identity. To be still recognizable by others, participants put pictures of their face on the virtual avatar’s chest.^3^ see for instance Fig. 1.

### Outline

This work is founded on two subsequent user studies based around the topic of visual (self-)representation using avatars. The relevance of our topic is introduced in Sect. 1, followed by an overview on different works that relate to our main topics in Sect. 2 and build the foundation for the hypotheses formulated in Sect. 3. To give an overview of the fidelity of current SVR platforms, we list different features of each platform in Table. 1 in Sect. 4.

In Sect. 5, we investigate in two directions: On the one hand, we investigate how different basic (default) avatars are perceived by the audience and on the other hand, we examine if and in how far one is able to create a representation of a given person using the standard customization tools of the respective platform.

The second study is presented in Sect. 6. Since almost all of the platforms also offer a desktop mode, we show how different devices (namely head-mounted display virtual reality devices and regular 2D monitors/screens) effect the perception of virtual avatars in regards to the uncanny valley effect.

For both studies we list the results in the associated Sections. In the end, we discuss their joint implications (Sect. 7) and limitations and also offer conclusions and an outlook in Sect. 8. In the following paragraph we list the main findings of both of the studies.

**Table 1:** Overview of social VR platforms and their avatar creation options. ?: Attribute could not be determined (platform still in closed-beta for example); N.A.: Not applicable, Assets and Age Control are only listed for platforms with configurators

Social VR platform	Desktop mode	Avatar creation	Assets	Age control	Bodyparts	Scaling	Tracking
	0 = No 1 = Yes	0 = No customization 1 = Upload 2 = Configure 3 = Generate	0 = None 1 = Glasses 2 = Tattoos 3 = Earings 4 = Prosthetics	0 = No 1 = Yes	1 = Head 2 = Torso 3 = Hands 4 = Upper body 5 = Full body	0 = No 1 = Yes	0 = Only basic 1 = Finger 2 = Lip sync 3 = Eye tracking 4 = Full body
AltspaceVR$^{\mathrm{a}}$	1	2	1	0	1,2,3	0	1,2,3
Bigscreen$^{\mathrm{b}}$	0	2	1	0	1,2,3	0	1
Facebook Horizon$^{\mathrm{c}}$	?	?	?	?	1,2,3,4	?	?
Glue$^{\mathrm{d}}$	1	2	1	0	1,3	0	1,2,3
MeetinVR$^{\mathrm{e}}$	1	2	1	0	1,3	0	2
Mozilla Hubs$^{\mathrm{f}}$	1	1	N.A.	N.A.	1,2,3	1	1,2
Neos VR$^{\mathrm{g}}$	0	1	N.A.	N.A.	1,2,3,4,5	1	1,2,3,4
RecRoom$^{\mathrm{h}}$	1	2	1	0	1,2,3	0	2
Sansar$^{\mathrm{i}}$	1	1,2	1,3	0	1,2,3,4,5	1	1,2,4
Sinespace$^{\mathrm{j}}$	1	1,2	2	0	1,2,3,4,5	1	2
Somnium Space$^{\mathrm{k}}$	1	1	N.A.	N.A.	1,2,3,4,5	1	1,2
Spatial$^{\mathrm{l}}$	1	3	N.A.	N.A.	1,2,3,4	0	1,2
The Wild$^{\mathrm{m}}$	1	0	0	0	1,2,3,4	1	0
VRChat$^{\mathrm{n}}$	1	1	N.A.	N.A.	1,2,3,4,5	1	1,2,4
vTIME$^{\mathrm{o}}$	0	2	1	1	1,2,3,4,5	0	1,2,3

$^{\mathrm{a}}$https://altvr.com/$^{\mathrm{b}}$https://www.bigscreenvr.com/$^{\mathrm{c}}$https://www.oculus.com/facebook-horizon/$^{\mathrm{d}}$https://glue.work/$^{\mathrm{e}}$https://www.meetinvr.com/$^{\mathrm{f}}$https://hubs.mozilla.com/$^{\mathrm{g}}$https://neos.com/$^{\mathrm{h}}$https://recroom.com/$^{\mathrm{i}}$https://www.sansar.com/$^{\mathrm{j}}$https://sine.space/$^{\mathrm{k}}$https://somniumspace.com/$^{\mathrm{l}}$https://spatial.io/$^{\mathrm{m}}$https://thewild.com/$^{\mathrm{n}}$https://hello.vrchat.com/$^{\mathrm{o}}$https://vtime.net/

## Related work - representation of humans and avatars

Virtual communication and collaboration uses in contrast to face-to-face encounters much more “mediated communication”. Typical available modes are audio-conferencing, video-conferencing and *computer-mediated communication* (CMC) [3]. One interface for CMC is provided by virtual reality technology [4]. Compared to face-to-face communication, *mediated communication* is significantly altered by the modifications and limitations of the communication channels available. Some nonverbal channels can be conveyed quite precisely, e.g. the auditory channel to convey speech or vocal utterances. On the other hand, aspects of *nonverbal communication* (NVC), such as physical appearance or facets of nonverbal behavior using the visual channel, can be transmitted better or worse dependent on technology and use-case (e.g. video conferencing vs. virtual world). This is essential to take into account as nonverbal communication is a central part in human social behavior, as it is able to express a lot what cannot be said adequately in words [5]. Nonverbal cues can lead to better coordination and improve understanding of the communication [6]. The absence of such nonverbal cues, like the loss of eye contact in video-conferencing applications, can therefore lead to reduced acceptance of video-conferencing technology, as people associate poor eye contact with deception [7].

Platforms using VR for interpersonal communication, interaction and collaboration are declared *social virtual reality* (SVR) platforms, as these distributed systems support mutual awareness and communication among their users [8]. Recently, a large number of SVR platforms have emerged that open another dimension of remote communication.

SVR platforms enable NVC to be conveyed by avatars (digital representatives with human controlled behavior for oneself or others) [9, 10]. The avatar’s appearance and behavior are actively interpreted throughout the communication process between the subjects [11]. These nonverbal communication cues are multifaceted which is why we restricted the focus of this paper to the aspect of the physical appearance and do not include other aspects of nonverbal behavior such as eye-contact, posture or facial expressions.

According to Mansour et al. the fidelity of the avatar’s appearance and behavior are a crucial factor on the perception of social interaction in SVR [12]. Their findings suggest that evaluating the effect of the behavioral fidelity on social interaction without taking the visual fidelity of the avatar into consideration (and vice versa) can lead to misleading results as the effects are interacting. Visual fidelity includes three properties that relate to the avatar’s appearance: Realism, Resemblance, and Personalization, and three properties that relate to the avatar’s behavior: Subtlety, Precision, and Expressiveness [11].

**Fig. 2:**
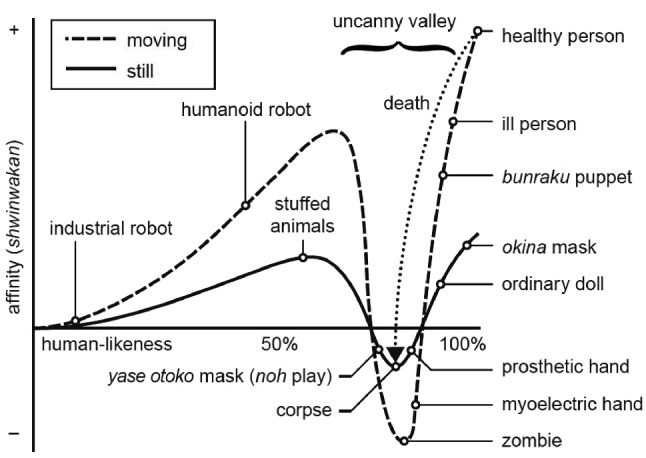
The original graph as proposed by Mori, from the 2012 translation [13]

Realism is also an aspect that must be regarded when considering a phenomenon known as the uncanny valley effect. This effect describes that the viewers’ acceptance of technically simulated entities is dependent on the behavioral and visual fidelity (realism) of the imagined carriers (such as avatars), but—contrary to what one might assume—does not show a linear relationship between the affinity for a virtual (humanlike) entity but a steep slope right before the relations match. Entities that fall into this slope are perceived as more eerie compared to less human like entities (see Fig. 2) [13].

Since the introduction of the uncanny valley in the 1970s to the field of robotics, it has been widely adopted to other fields in numerous studies: for actually built robots [14], 2D representation of persons in film [15, 16] as well as in computer games [17, 18], and even for cats [19]. All those studies, except for the actually built robots, have in common that they were carried out using a 2D output device such as a monitor or a projector, but more immersive technologies such as stereoscopic displays or HMDs have not been given much attention so far. However, there are indications that HMD VR in comparison to 2D monitors elicit stronger negative emotions [20], which would eventually pronounce the UV effect. Its lack of investigation is in particular surprising given the fact, that the representation of avatars and in particular the self-representation plays a critical role in HMD based virtual environments (especially for those with a focus on interpersonal communications, such as mentioned above) and is closely linked to embodiment and immersion [21].

As one of the first to mention the UV in context of *Immersive Virtual Environments* (IVE), Lugrin et al. studied the “*illusion of virtual body ownership*”. They found that being represented by a more human like virtual body leads to a higher feeling of having two separate bodies then when a person was represented by a more robot like body [22]. This led them to the conclusion, that this might be due to the same reasons as stated for the uncanny valley effect on monitors. They mention that participants were more concerned about details such as a correct arm length when their body was represented by a virtual human as when it was rendered as a virtual robot. Schwind et al. examined the effect of different hand styles in VR. They found that, while both men and women do feel discomfort using hands of the other gender, “*an overall decrease of presence or likability as predicted by the UV was not found*” [23].

Latoschik et al. studied the effect of avatar realism on embodiment and social interactions in VR. Their data indicate that realistic avatars were rated significantly higher more human like than abstract wooden mannequin avatars but also suggests potential UV. However, all their investigations only took place in VR. There are no data or studies on how the measured effect is enhanced or diminished when transitioning from a 2D to a 3D environment [24].

Maloney et al. studied differences in NVC between IVEs, traditional 2D/3D virtual worlds and reality. Their findings suggest that the uniqueness of nonverbal communication in social VR lies in its increased effectiveness for conveying meaning compared to traditional virtual worlds, and in its flexibility and less awkwardness compared to the offline world [25].

Bailenson et al. investigated proxemics between avatars in VR considering personal space and mutual gaze in intimacy-equilibrium theory. Intimacy-equilibrium theory was first introduced by Argyle et al. and states that people move toward an equilibrium level of “intimacy” with others, where intimacy is a function of physical proximity, eye contact, facial expression (smile), topic of conversation (how personal), tone of voice (warm), and so on [26]. The result of their studies provides proof that nonverbal social norms regarding proxemics are consistent in virtual and real physical environments [27]. However, a comparison between 2D and 3D was yet not taken into account. Susindar et al. investigated the elicitation of emotions in VR and compared it with a regular screen [28]. In their study, negative emotionally-charged stimuli (fear and anger) were introduced via two different screen configurations (desktop computer and VR) and were evaluated based on the performance in a decision making task. The result showed that the influence of the target emotions on the decision-making behavior was more pronounced in the VR condition than the desktop condition. Therefore, it can be derived that the usage of IVEs leads to more effective emotion generation than the use of less immersive presentation methods such as common screens. Villani et al. found a link between presence and emotions in VR. They figured out that the measured level of presence was significantly higher in anxious virtual environments than in relaxing ones and therefore triggered a stronger emotional response [29].

As all these detailed facets play a vital role in the complex field of NVC, it is worthwhile to investigate how current SVR platforms address the challenge of implementing self-representation and self-expression using avatars. Focused on nonverbal behavior and behavioral fidelity, Tanenbaum et al. developed an inventory of nonverbal communication in commercial SVR platforms, to get a deeper understanding of the design strategies for expressive NVC used [30]. They identified dominant design strategies for movement, facial control, and gesture in commercial VR applications but remarked a paucity of interaction paradigms for facial expression and the almost nonexistence of meaningful control over aspects of nonverbal communication such as posture, pose, and social status. However, they did not take into account visual fidelity with its aspects of realism and resemblance.

In summary, while there has been done a considerable amount of research regarding the UV in 2D environments, investigations of 3D environments are lacking. Those investigations are, however, important as due to the special and different characteristics of IVE—some of these findings cannot be taken for granted or simply taken over, but have to be (re)evaluated. Also topics such as interactions might be prone to fall into the UV [31]. In addition, current literature is missing an overview of how people can represent themselves within a SVR application in a way that they can be recognized by others.

Therefore, in this context, we make two contributions that consider the visual appearance of avatars in SVR platforms. First, we take inventory of selected commercial SVR platforms in terms of various aspects and further focus on the visual self-representation and the resemblance of the avatars to the depicted subject. Second, we explore the UV effect regarding the visual representation of the avatar in VR compared to traditional 2D screens.

## Hypothesis

As we have seen there exist various possibilities and differences in the way avatars can be created, viewed and also perceived.

There seem to be large differences in the visual fidelity of avatars across platforms. It is expected that customized avatars allow a clear recognition of the represented person. Furthermore, we do not expect a difference between pre-given and customized avatar for the variables human likeness, eeriness and likability.

To investigate if those possibilities cause differences in the perception of avatars we pose the following hypothesis:

H1.1: The **resemblance** of a **custom avatar** with the **represented person** is **given**.
H1.2: The **resemblance of an avatar is independent** from individual parameters e.g. likability or age.
H2.1: There is **no difference in perceived human likeness** between custom and default avatars.
H2.2: There is **no difference in perceived eeriness** between custom and default avatars.
H2.3: There is **no difference in perceived likability** between custom and default avatars.
H3.1: There is **no difference in perceived human likeness** of characters in regards to the different output devices HMD and monitor.
H3.2: There is **no difference in perceived eeriness** of characters in regards to the different device types HMD and monitor.
H3.3: There is **no difference in perceived likability** of characters in regards to the different device types HMD and monitor.

To answer the posed hypotheses we have undertaken two user studies.

## Avatar creation and customization

As stated in Sect. 1, there are many different SVR platforms today that aim on enabling users to collaborate with each other in VR in some way. Since those platforms do not have a single target audience but aim to fulfill a special need within a domain (some focus on entertainment while others focus more on business), they come with different feature sets but have im common that there always is the need to visualize how users are represented within a world and how users can interact with each other. It should be noted that users do not always want to represent themselves according to reality, as is the case with some recreational platforms such as VRChat or Neos VR. As one of few, Kolesnichenko et al. interviewed industry experts on their opinion in regard to avatar identification and representation. They state that the context in which users are represented influences the way people want to be represented and that their preferred representation is not limited to one avatar but several avatars for different situations [32]. For this work, our focus is to constitute the different possibilities of personal avatar creation in SVR platforms and the accompanying possibilities of realistic self-representation and nonverbal communication. Furthermore, we want to review the variables related to the UV in context of user created avatars used on SVR platforms. The selection process was driven by the want to cover a wide mix of platforms, taking into account popularity, target audience, use case, and most importantly, to cover different fidelity levels of avatar appearance. The platforms range from very popular, as for example VRChat or RecRoom, to services with smaller user base. However, if evident that a platform might not be maintained anymore, it was excluded.

### Platform features

Across all platforms, we were particularly interested in whether and to what extent it is possible to create an individualized avatar that matches the appearance of the controlling person. Therefore, we investigated the selected SVR platforms regarding the following list of attributes which allow the platform user to represent various information via his/her avatar in the virtual space. When investigating, we either tested the application, or in cases where this was not possible, we relied on official documentation or videos. The examined attributes are displayed in Table 1 and described next.

#### Desktop mode

Valid if the platform has an option for users to participate in the virtual space via a 2D monitor.

#### Avatar creation

Possibility to customize or create an avatar based on user preferences.

- *Upload:* Option to upload a custom created avatar to the platform.
- *Configure:* Alteration of a standard avatar with a pre-defined set of applicable variations such as hairstyle, eye color, size, skin and clothing parts.
- *Generate:* Generating the avatar from a picture of the user.

#### Assets

Customization possibilities, such as glasses, tattoos, earings, prosthetics, amputations or wheel chairs, which contribute to the visual resemblance and support the user in identifying with the avatar. Assets are only tracked for avatars created via configurators, as the creation of custom avatars is platform unrelated.

#### Age control

Option to adjust age-related features exceeding attributes such as hair color. This is limited to configured avatars, as the creation of custom avatars is not related to the platform in use.

#### Body parts

Indicates if the avatar is displayed as a full body or whether the body is only partially shown, for example in a combination of a floating head and hands. Having a full-body avatar instead of just a head lets you visualize more information such as body shape and size or other individual parameters such as clothing preferences and prosthetics. This attribute is limited to configured or generated avatars.

#### Scaling

The possibility to scale the avatar to visualize different body sizes.

#### Tracking

Tracking is essential to represent movement and gestures, which depending on the respective culture, can be a supplement in communication. We classified tracking advancing over the basic VR setup tracking (head and hands) into four categories which differ in the technology used. These tracking methods can only be used if the user is in possession of the appropriate hardware.

- *Full Body Tracking:* Tracking and mapping the position of extremities such as arms, legs, and feet in real time. This can be achieved by using additional sensors such as Vive Trackers, Xsens or an Azure Kinect.
- *Finger Tracking:* Mapping of distinct finger positions onto the avatar with the use of a camera or in a simplified version due to the finger position on the controllers for example with the Oculus Touch controllers or Leap Motion.
- *Eye Tracking:* Nowadays, eye-tracking technology is available as hardware for 2D monitor setups as well as for some HMD VR headsets such as the HTC Vive Pro Eye or the Varjo VR-3.
- *Lip Sync:* Matching the lip movement according to the spoken vocals.

## Study 1 - differences in perception according to customization

To further extend our overview of SVR platforms, we examine and compare the selected platforms (Table 1) in terms of the user’s visual representation. Thus, we conducted a study to investigate and compare different popular SVR platforms with the regard to the visual representation of their avatars.

However, the strict COVID-19 restrictions did not allow us to run user studies with HMDs. Thus, we had to exclude this condition and limit the study to on-screen representations. Because the resemblance of an avatar to the real person to be depicted is crucial for self-presentation and self-expression in SVR, data generation also focused on this objective. It also allowed us to compare default avatars with customized avatars, taking into account interesting attributes such as eeriness or likability.

### Participants

Between 23rd of December 2020 and 4th of January 2021 a total of 109 participants could be recruited via multiple public social channels, such as Reddit, Discord or Facebook for a within-subject test population. The participants ranged in age from 13 to 61 ($\bar{x}=30.1, SD=9.3$).^4^ Almost half the participants stated they played video games for more than 10 hours per week (45.0%), 26.6% 5–10h, 14.7% 1–5 hours, and 13.8% played less than 1 hour per week.

### Procedure

We used the online-survey tool called SoSci Survey (www.soscisurvey.de) to conduct the survey. Each participant could take part in the survey using a common web browser on a 2D monitor or a smartphone.

**Fig. 3:**
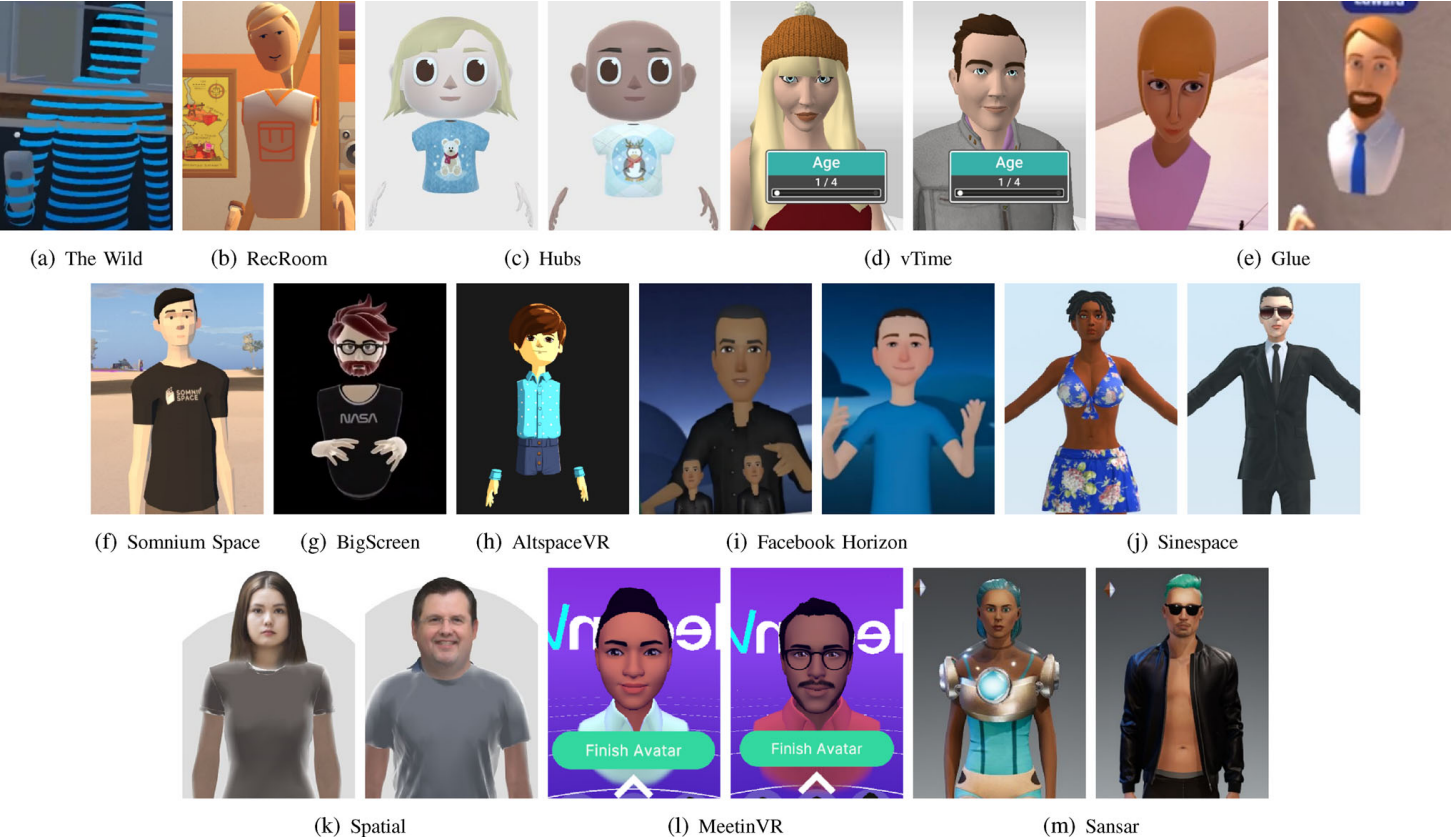
Default/Representative characters of evaluated platforms. Sorted by mean humanlikeness, low–high

**Fig. 4:**
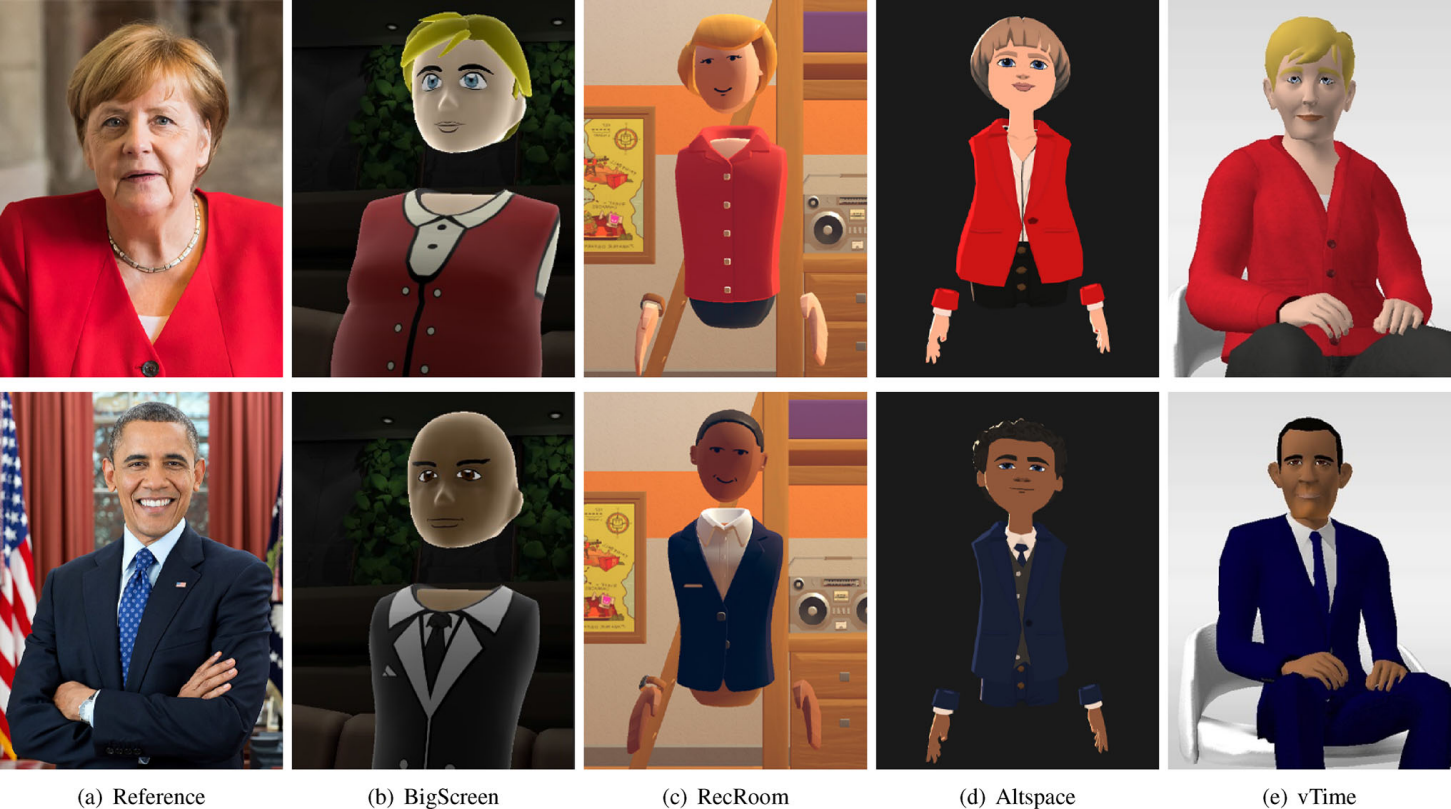
Angela Merkel (top row) and Barack Obama (bottom row) avatars

**Table 2:** Overview of the selected platforms ordered by human likeness

#	Platform	Likability		Eeriness		Human likeness		Style		Freely available	Avatar customization
		$\bar{x}$	SD	$\bar{x}$	SD	$\bar{x}$	SD	$\bar{x}$	SD		
(a)	The Wild	2.17	1.90	3.74	2.40	2.08	1.87	1.72	1.22	no	0
(b)	RecRoom	3.79	1.85	2.85	1.80	3.17	1.88	1.52	0.83	yes	2
(c)	Hubs	3.23	1.90	3.72	2.03	3.40	1.96	1.35	0.55	no	4
(d)	vTime	2.39	1.35	4.65	1.81	3.94	2.08	2.92	1.30	yes	2
(e)	Glue	3.27	1.58	3.83	1.90	4.24	2.16	2.68	1.33	no	4
(f)	Somnium Space	3.53	1.69	3.17	1.57	4.27	2.34	2.48	1.34	yes	3
(g)	BigScreen	4.03	1.60	2.87	1.63	4.55	1.95	2.46	1.19	yes	2
(h)	Altspace	4.33	1.59	2.79	1.63	4.89	2.14	2.04	1.15	yes	2
(i)	Horizon	4.06	1.53	3.05	1.78	5.17	2.01	2.89	1.37	no	4
(j)	SineSpace	3.79	1.63	3.45	1.83	5.57	2.14	4.13	1.41	yes	1,3
(k)	Spatial	2.72	1.62	5.02	2.03	6.06	2.68	5.60	1.23	yes	0
(l)	MeetInVR	4.85	1.35	2.37	1.37	6.40	1.85	4.52	1.44	no	4
(m)	Sansar	4.45	1.69	2.64	1.52	6.63	2.06	5.34	1.34	yes	1

Eeriness and likability were measured on a scale from 1–7; human likeness on a Scale from 1–9; style (cartoonish to visually detailed) on a scale from 1–7. No. # shows the avatar label as listed in Fig 3. Avatar customization: 0: No customization possible; 1: Editor with limited customization (e.g only extendable with micro-transactions or other procedures); 2: Editor with rich customization options; 3: No Editor, free customization; 4: No information

**Table 3:** Overview of the selected platforms considering custom avatar creation

#	Platform	Custom Avatar	Likability		Eeriness		Human Likeness		Resemblance	
			$\bar{x}$	SD	$\bar{x}$	SD	$\bar{x}$	SD	$\bar{x}$	SD
(b1)	BigScreen	Angela Merkel	2.23	1.35	4.15	2.06	2.92	1.53	1.73	1.25
(b2)	BigScreen	Barack Obama	2.61	1.38	3.70	2.10	2.94	1.57	1.70	1.32
(c1)	RecRoom	Angela Merkel	3.66	1.70	2.93	1.83	3.56	2.05	3.78	2.59
(c2)	RecRoom	Barack Obama	3.62	1.68	2.89	1.82	3.72	1.99	3.38	2.36
(d1)	Altspace	Angela Merkel	3.81	1.64	2.77	1.68	4.67	1.87	3.59	2.37
(d2)	Altspace	Barack Obama	4.05	1.48	2.60	1.50	4.57	1.98	2.07	1.34
(e1)	vTime	Angela Merkel	2.26	1.28	4.56	1.94	4.11	2.15	3.39	2.21
(e2)	vTime	Barack Obama	3.48	1.51	3.66	1.71	4.88	1.87	5.29	2.35

Eeriness and likability were measured on a scale from 1–7; human likeness on a Scale from 1–9; resemblance (not to total) on a scale from 1–10. No. # shows the avatar label as listed in Fig. 4

We considered a total of 15 SVR platforms, but had to exclude 2 platforms from our survey as those platforms did not provide an avatar configuration option but only the option to upload own avatars (VRChat and Neos VR). The unrestricted creation of full custom avatars using 3rd party tools disables defining general attributes of the platform’s avatars.

We selected the default avatars presented by the platform owners of the remaining 13 SVR platforms included in the study, displaying both genders whenever possible (8 platforms) resulting in a total of 21 avatars, see Fig. 3. In addition to the 21 default avatars of the SVR platforms considered, we created eight additional avatars (Angela Merkel, Barack Obama, Fig. 4). We chose these two public figures because we assumed that they are well known and thus should be recognizable by all participants. The avatars of Merkel and Obama were created for four platforms (Altspace, Bigscreen, RecRoom, vTime), all by a single person, with average modeling skills, who has not been using any of the platforms before. We had to exclude the remaining platforms because avatar customization was either not freely accessible or no suitable customization could be achieved.

We investigated the commonly used variables human likeness, likability and eeriness to compare the results of our two studies later on (Sect. 6). Additionally to the 9-point scale for human likeness from *“very mechanical”* to *very humanlike”* and the 7-point scales for eeriness and likability we added a new 7-point scale variable from *“cartoonish”* to *“visually detailed”* to rate the avatar styles. To investigate the degree of the possible visual self-representation the participants were shown half-body portrait pictures of the politicians. The participants were asked to state which person was shown on the pictures by clicking on one of the pre-defined answers. In the following, the participants had to rate the resemblance of the virtual avatars with the two politicians for each of the four platforms on a 10-point Likert scale ranging from *“not”* to *“total”* resemblance.

### Results

In Table 2 is displayed how the different avatars representing a platform were perceived regarding the attributes asked. Sorted by the average human likeness measured, a big variety ranging from 2.08 for platform The Wild to 6.63 for platform Sansar shows up. The overall human likeness was 4.64. The character style, ranging from cartoonish to visually detailed, was perceived between 1.35 in Hubs and 5.34 in Sansar. The overall character style was 3.0, which means it was perceived more cartoonish in average. Likability and Eeriness were perceived less various. The overall likability was 3.59 and the overall eeriness 3.39.

While Angela Merkel was recognized by 93.6% of the participants, Barack Obama achieved 100%. We examined the perceived resemblance of Angela Merkel’s and Barack Obama’s avatars to the real persons for the four platforms chosen. One of the main hypotheses (H1.1) states that the resemblance of oneself to a custom avatar, created with an avatar configurator to resemble oneself, is given (Table 3).

A single-sample *t*-test was conducted to investigate the degree of resemblance for the custom avatar of Merkel and Obama. For Merkel, there was a significant difference in the scores for resemblance compared to the *“resemblance-threshold”* ($\bar{x}=3.12, SD=1.51, T(108)=-12.94, p<.001$). For Obama, there was a significant difference in the scores for resemblance compared to *”resemblance-threshold”* ($\bar{x}=3.11, SD=1.29, T(108)=-15.19,~ p<.001$).

Thus, we consider that the avatars did not resemble the public figures represented good enough to be recognized. Hence, **H1.1 [The resemblance of a custom avatar with the represented person is given] cannot be confirmed and has to be rejected**. Resemblance was the worst in BigScreen for both Obama ($\bar{x}=1.7, SD=1.32$) and Merkel ($\bar{x}=1.73, SD=1.25$) and the best in vTime for Obama ($\bar{x}=5.29, SD=2.21$) and in RecRoom for Merkel ($\bar{x}=3.78, SD=2.59$).

**Table 4:** Multiple regression was calculated based on the independent variables “Age”, “Human Likeness”, “Likability” and “Eeriness” for “Resemblance” as the dependent variable for all 4 platforms evaluated in this work as well as over all platforms

	Altspace			BigScreen			RecRoom			vTime			Overall		
	b	95% CI		b	95% CI		b	95% CI		b	95% CI		b	95% CI	
(Intercept)	1.11	[−0.23; 2.45]		3.37	[ 1.76; 4.97]*		0.88	[ 0.01; 1.76]*		2.74	[ 1.05; 4.42]*		1.23	[ 1.05; 4.42]*	
Age	$$ -$$0.04	[−0.07; −0.01]*		$$ -$$0.04	[−0.08; −0.01]*		−0.03	[−0.04; −0.01]*		−0.07	[−0.10; −0.04]*		$$ -$$0.04	[−0.10; −0.04]*	
Human Likeness	0.07	[−0.09; 0.23]		0.10	[−0.12; 0.33]		0.13	[ 0.04; 0.22]*		0.44	[ 0.27; 0.61]*		0.21	[ 0.27; 0.61]*	
Likability	0.49	[ 0.32; 0.66]*		0.47	[ 0.27; 0.66]*		0.26	[ 0.15; 0.37]*		0.44	[ 0.24; 0.65]*		0.43	[ 0.24; 0.65]*	
Eeriness	0.34	[ 0.17; 0.52]*		−0.04	[−0.20; 0.11]		0.15	[ 0.05; 0.24]*		0.00	[−0.17; 0.17]		0.24	[−0.17; 0.17]*	
R$^2$	0.21			0.13			0.18			0.31			0.18		
Adj. R$^2$	0.19			0.12			0.17			0.29			0.18		

$^*$0 outside the confidence interval

**Fig. 5:**
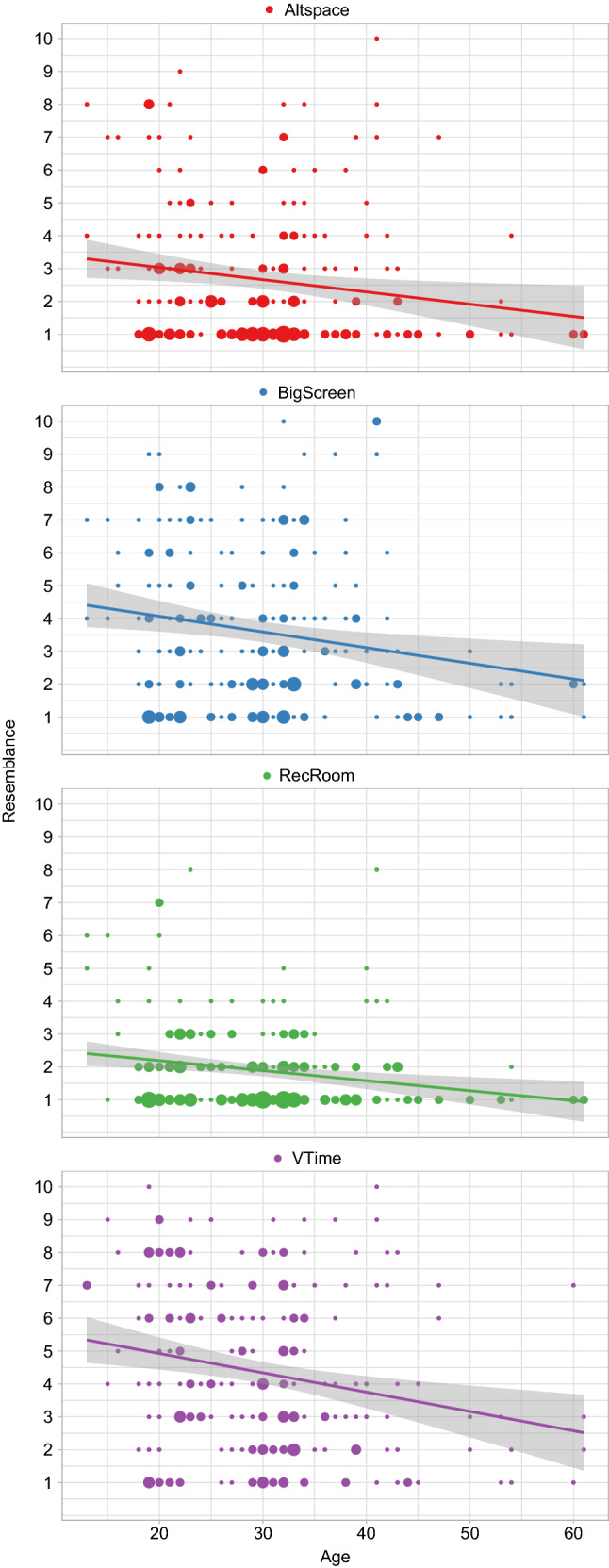
Scatter Plot for all platforms tested showing “Age” on the x-axis and “Resemblance” on the y-axis. Increased dot-size shows a higher number of observations at this point

To further narrow down what might be a predictor of resemblance, a multiple regression was calculated based on the independent variables “Age”, “Human Likeness”, “Likability” and “Eeriness” (Results are summarized in Table 4). The regression was done for all different platforms investigated as well as over all platforms. We can see that for all platforms, an increase in age by 1 year, declines resemblance by 0.04 (standardized small effect $r=-0.19$). A scatter plot of the relation is shown in Fig. 5.^5^ With the different models we can explain 21% Altspace, 13% BigScreen, 18% RecRoom and 31% vTime of the variances for the dependent variable resemblance. Likability also is significant over all platforms. Overall, an increase in likability would increase resemblance by 0.43 (standardized small to medium effect $r=0.38$). Given the fact, that 2 of the 4 tested independent variables (likability and age) are significant not only when viewed overall platforms, but also for all the platforms tested individually, we see that those parameters do predict resemblance to a certain degree. Therefore, **H1.2 [The resemblance of an avatar is independent from individual parameters e.g. likability or age] cannot be confirmed and has to be rejected**.

Considering human likeness, eeriness and likability, we can say that both, Obama and Merkel were in average perceived about equally human like for all platforms. Avatars in BigScreen were in average found the least human like (Obama: 2.87, Merkel: 2.85). Merkel was found most human like in Altspace (4.64) and Obama in vTime (4.65). Obama and Merkel were rated similarly likable on all platforms with BigScreen rated the worst (Obama: 2.52, Merkel: 2.19) and Altspace the best (Obama: 3.95, Merkel: 3.81). The perceived eeriness was highest in Altspace for Merkel (4.52) and in BigScreen for Obama (3.69) and lowest for both in Altspace (Obama: 2.52, Merkel: 2.75).

Furthermore, we wanted to investigate whether our customized avatars (Merkel, Obama) were perceived differently from the default avatars regarding human likeness, eeriness, and likability. For this purpose, we averaged the values of Merkel and Obama and declared them “custom avatars”. Then, we compared the custom avatars with the default avatars for all four platforms using ANOVA. For human likeness we found significant differences between the default and the custom avatars in BigScreen ($F=60.17, p<.001$) and in RecRoom ($F= 5.55, p=.019$). As there are platform dependent variations, hypothesis **H2.1. [There is no difference in perceived human likeness between custom and default avatars.] cannot be confirmed and has to be rejected**. Concerning eeriness we also found significant differences between the default and the custom avatar in BigScreen ($F= 16.94, p<.001$) and in VTime ($F= 7.51, p=.007$). Therefore, hypothesis **H2.2 [There is no difference in perceived eeriness between custom and default avatars.] cannot be confirmed and has to be rejected**, as there are platform dependent differences, too. Significant differences were also found for likability in BigScreen ($F=74.02, p<.001$) and in vTime ($F=8.09, p=.005$). Thus, also hypothesis **H2.3 [There is no difference in perceived likability between custom and default avatars.] cannot be confirmed and has to be rejected**. Although significance was found for human likeness, eeriness and likability in BigScreen, for human likeness in RecRoom and for likability in vTime, Altspace remained insignificant. Therefore, we conclude that differences in the perception of default and custom avatars are generally a platform-dependent matter.

## Study 2 - differences in perception according to the display type

**Fig. 6:**
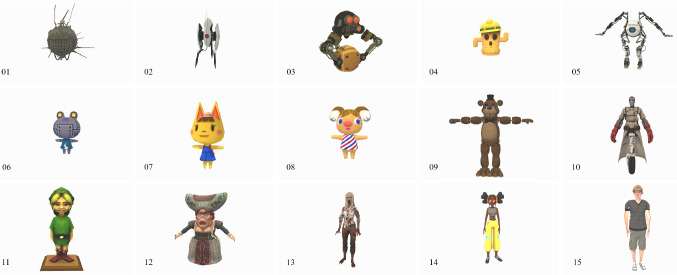
All different characters used for evaluation. 1. Eyebot, 2. Turret, 3. JRRobo, 4. Lloyd, 5. Atlas, 6. Ribbot, 7. Katie, 8. Alice, 9. Freddy, 10. Medic, 11. Link, 12. Dutchess, 13. Zombie, 14. MixamoGirl, 15. Remy

As shown in Sect. 2 it exists a number of works related to the UV but only a few works has taken more immersive technologies such as stereoscopic displays or HMDs into account. These technologies not only enable a new way of experiencing content, there are also signs that indicate a more pronounced effect regarding emotions. Due to this, we compare the UV effect between regular 2D monitors and head-mounted VR for 15 different characters, see Fig. 6.

### Terminology

In this section, we compare and list research from different domains. Unfortunately, the use of terminology is not consistently used between domains. For the scope of this publication, we use the terminology “monitor” when referring to technology that otherwise might be also addressed as a 2D screen [33], display [34, 35], or desktop [20, 36]. Also we use “character” as a terminology that does not distinguish between the often used definitions for (human-controlled) *avatars* or (computer-controlled) *agents* since the findings of our research apply to both of them. The term HMD refers to a terminology otherwise referred to as VR-HMD but must not be confused with an HMD used for augmented reality such as the Microsoft HoloLens.^6^

### Test environment

We used the game engine Unity^7^ to create a test environment containing two cameras, one simulating the viewport using a monitor and one simulating the viewport of the HMD. As Schneider expressed concern that the background influences the way characters are perceived  [18], we decided not to use any background pictures, just as he did, and instead showed the character models in front of a neutral gray background. Due to the nature of this study, displaying the characters in flat mediums like video clips or pictures as done in earlier research [18, 37] would have been counterproductive as they would generate a different experience compared to the depth of an actual 3D model.

### Setup

The monitor tests were conducted with a regular computer monitor with 1920$\times$1080px resolution. For the immersive environment, a HTC Vive HMD was used.

#### Virtual setup

The 15 virtual characters used in the study were shown to each participant from within a unity application. They were presented in a randomized order to minimize potential bias through the sequence of models

On the press of a button, one character disappeared and the next character was shown in its place. Every character has been resized to fit into the same field of view from a similar distance. For orientation, the camera had been placed above a light-gray rectangular platform with a blue arrow pointing towards the direction of the models. Camera rotation was possible both in the HMD (by rotating the head) and the monitor (by using a computer mouse), however movement was not implemented.

#### Character setup

It was important to select characters that distribute equally on the human likeness scale. When choosing models, we looked to find enough to cover all of Mori’s original curve. We looked for models within three categories: “*clearly robotic*”, “*clearly human*” and “*in-between*” to the best of our judgment. To cover the whole spectrum, we selected from two resources: VG-Resource^8^ and Adobe$^{\mathrm{TM}}$ Mixamo.^9^ The final set of characters is shown in Fig. 6. All models are textured rigid bodies, rendered in neutral Pose or T-pose without animations. Bartneck et al. made an additional note in regards to anthropomorphism “*Knowing that a certain entity is a robot or human does in itself not constitute a positive or negative effect on its likability or human likeness. Instead, the appearance of the entity is mainly responsible for its likability.*” [38].

**Fig. 7:**
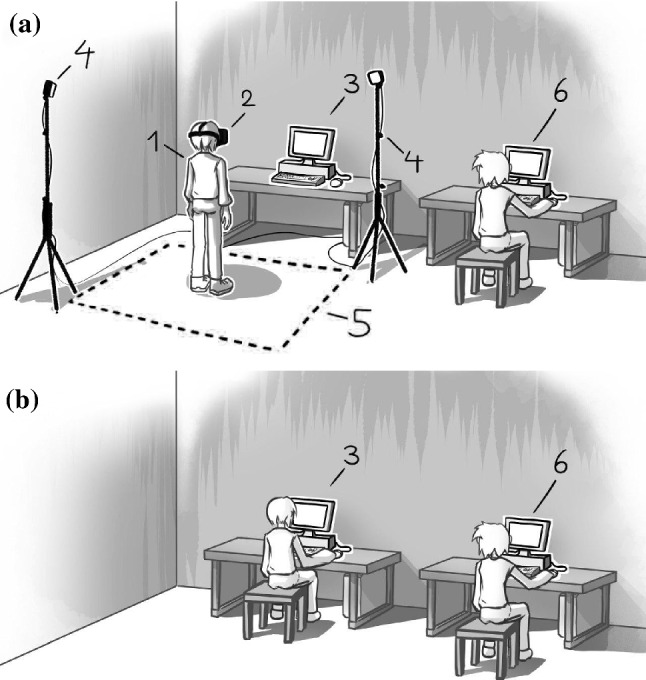
Drawing of the a) HMD setup b) monitor setup. 1: participant wearing the HMD; 2: HTC VIVE HMD; 3a): computer used for running HMD simulation 3b) participant sitting at monitor test setup; 4: HTC Vive base stations; 5: HMD area; 6: test operator asks and fills in questions from questionnaire

### Evaluation

Tests were conducted in the time-span from 10th of February 2020 to 15th of February 2020. The between-group test population consisted of a total of 27 participants, split into 14 people for the HMD and 13 for the monitor. All attendants except one were students in their 20s (19 to 28 years old; $\bar{x} = 23.1; SD = 2.5$). The majority stated they did play video games (85%). Participants were asked to rate the 15 characters according to human likeness, eeriness, and likability. In Mori’s original article, no practical approach to empirically study the topic was given. But later studies have mainly been using eeriness and attractiveness, or eeriness and likability on Likert-scales with varying ranges to determine the UV, however these parameters are not without criticism. For comparison reasons, we decided to stick with the more commonly used variables human likeness, likability, and eeriness. Furthermore, participants needed a self-report tool with sliders to express their opinion. These were given similar to these of MacDorman’s revised variables [37]. This however was not adequate for the HMD test group, as filling out the form manually and simultaneously looking at the character would break the immersion. An explanation of the range of the scales and the test procedure was given previous to randomly assigning the participants to use either the HMD or the monitor. During the tests, participants were asked verbally by the test operator where on the scales they perceived the virtual character to be. This verbally given answer was then noted by the operator, as seen in Fig. 7.

#### Questionnaire

Before taking part in the survey, participants were told about the test procedure. They were informed that their data was used for this survey and we asked for their consent. Naturally, they were also told they could stop test procedures at any time, to which consent had to be given on a digital form. After recording the variables, the participants were asked to fill out the demographic data such as age and gender in private.

#### Investigated variables

The scientific literature about the UV shows large variations regarding the variables and methods used for measuring the UV [37, 39–41]. Bartneck et al. use a 7-point semantic differential scales to measure the likability and human likeness [38]. The original 1970 essay from Mori was written in Japanese and presented only his hypothesis while not accompanying a clear testing method. Mori uses the term 
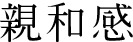
 which consists of the part 
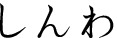
 “*shinwa*” which means *friendship*, *fellowship*, or *amicability* and 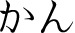 “*kan*” which stands for *feeling* or *emotion*. This was refered to *energy* in the first translation which was later changed to *familiarity*. However, the term *familiarity* posed problems, as “*[...] it is incoherent to conceive of the dependent variable simply as familiarity, because the zero crossing in the graph is a total novelty, and an entity cannot cross below total novelty into negative familiarity.*” [42]. The 2012 translation for the IEEE contained a revision by MacDorman, where it was labeled as affinity. Different works show various translations of *familiarity*. Ho and MacDorman have found *shinwakan* to be variously translated as familiarity, affinity, comfort level, likability, and rapport [43]. Furthermore, early results to MacDorman’s research “*[...] indicate that the perceived human likeness of a robot is not the only factor determining the perceived familiarity, strangeness, or eeriness of the robot. This suggests that other factors could be manipulated to vary the familiarity, strangeness, or eeriness of a robot independently of its human likeness.*” [39]. Accounting for these reasons, we decided to use following terms as dependent variables: human likeness, eeriness, and likability. This study’s independent variables were the output devices “monitor” and “HMD”.

### Results

**Fig. 8:**
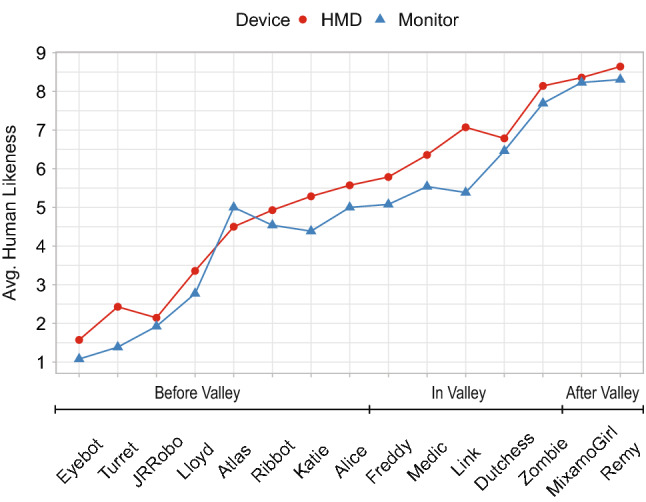
Graph showing the perceived human likeness of all evaluated 3D characters per device on a scale from 1 (very mechanical) - 9 (very human like)

**Fig. 9:**
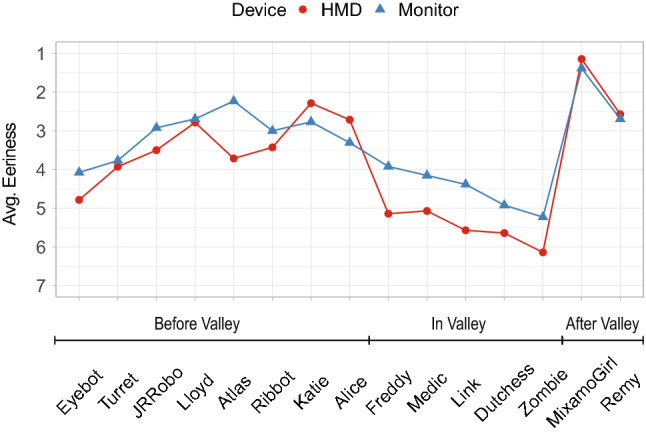
Graph showing the average values for perceived eeriness for each 3D character per device on a scale from 1-7

**Fig. 10:**
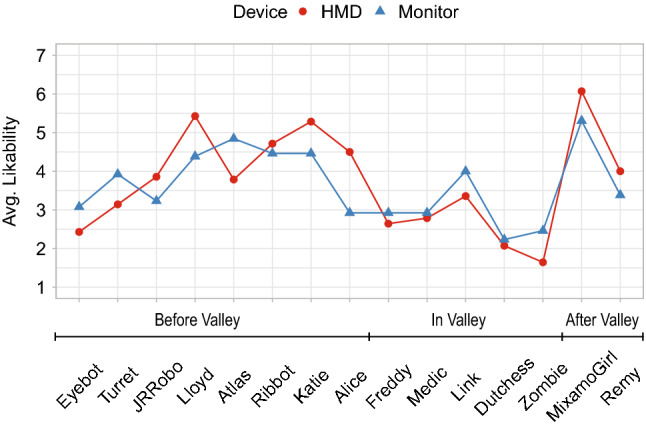
Graph showing the average values for perceived likability for each 3D character per device on a scale from 1–7

The choice of the 3D characters resembles an almost linear increase in human likeness (see Fig. 8) and is considered essential for further examinations on the UV effect in regards to eeriness (see Fig. 9) and likability (see Fig. 10). Similar to Schneider, the characters were placed according to their average human likeness score [18].^10^ Section 2 showed that there is a respectable amount of literature indicating differences in the perception of virtual content depending on the chosen output device such as an HMD or monitor. We used ANOVA to check if there are significant differences in perceived eeriness, human likeness, and likability induced by the device used. Plotting the results on the respective graphs revealed especially large differences of the average eeriness between HMD and the monitor within the UV. Consequently, to investigate the differences individually, we separated the results—following the literature—into following three parts: before the valley, within the valley, and after the valley. A more detailed discussion is presented in [2].

#### Differences in perceived human likeness per device

Comparing the means of dependent variable “*human likeness*” having the device-types “*HMD*” and “*monitor*” as an independent variable over all different participants with an ANOVA, reveals **a significant difference with** $$F(1, 403) = 4.87$$, $$ MSE = 6.15$$, $$p = .028$$.

Further subdividing the data set into the three parts *before valley*, *in valley*, and *after valley* to investigate them independently reveals that in *before valley* there is **no significant difference with** $$F(1, 214) = 3.11$$, $$ MSE = 3.73$$, $$p = .079$$, in *in valley* there is **a significant difference with** $$F(1, 133) = 10.42$$, $$ MSE = 2.06$$, $$p = .002$$, in *after valley* there is **no significant difference with** $$F(1, 52) = 1.86$$, $$ MSE = 0.39$$, $$p = .179$$.

We could show, that the perception of human likeness is dependent on the output device especially for characters within the uncanny valley. Therefore, we **reject hypothesis H3.1 [There is no difference in perceived human likeness of characters in regards to the different output devices HMD and monitor]**.

#### Differences in perceived eeriness per device

Comparing the means of dependent variable “*eeriness*” having the device types *HMD* and *monitor* as an independent variable over all different participants with an ANOVA reveals **a significant difference with** $$F(1, 403) = 6.20$$, $$ MSE = 3.52$$, $$p = .013$$.

Subdividing the data set as before reveals that in *before valley* there is **no significant difference with** $$F(1, 214) = 1.67$$, $$ MSE = 2.84$$, $$p = .197$$, in *in valley* there is **a significant difference with** $$F(1, 133) = 18.37$$, $$ MSE = 1.80$$, $$p < .001$$, in *after valley* there is **no significant difference with** $$F(1, 52) = 0.23$$, $$ MSE = 1.97$$, $$p = .637$$.

Again, we can see that the influence of different output devices (HMD and monitor) on the perception of eeriness is stronger within the UV compared to other parts of the curve. Consequently, we **reject hypothesis H3.2 [There is no difference in perceived eeriness of characters in regards to the different device types HMD and monitor]** for within the UV because there is a difference in perceived eeriness.

#### Differences in perceived likability per device

Comparing the means of dependent variable “*likability*” having the device-types *HMD* and *monitor* as an independent variable over all different participants with an ANOVA shows **no significant difference with** $$F(1, 403) = 0.18$$, $$ MSE = 3.36$$, $$p = .668$$.

Separating the data set as before reveals that in *before valley* there is **no significant difference with** $$F(1, 214) = 0.90$$, $$ MSE = 3.16$$, $$p = .344$$, in *in valley* there is **no significant difference with** $$F(1, 133) = 2.41$$, $$ MSE = 2.32$$, $$p = .123$$, in *after valley* there is **no significant difference with** $$F(1, 52) = 2.33$$, $$ MSE = 2.75$$, $$p = .133$$.

We could not find a significant difference between the used output device for the variable *likability*, hence stated hypothesis **H3.3 [There is no difference in perceived likability of characters in regards to the different device types HMD and monitor] can not be rejected and holds true **.

#### Correlation between likability and eeriness

**Fig. 11:**
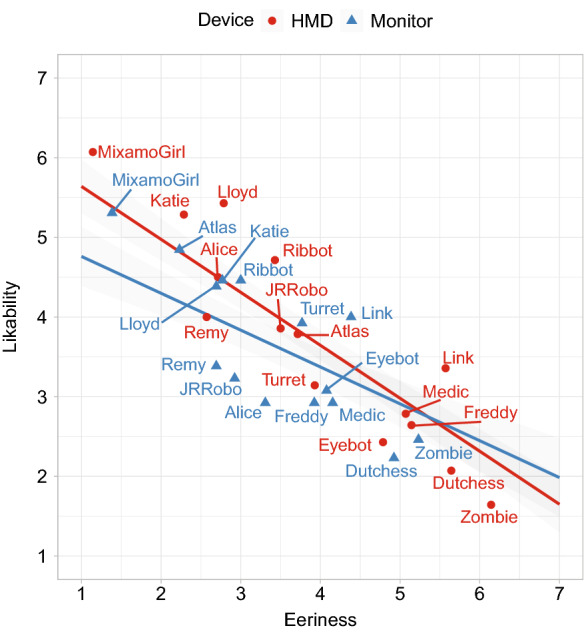
Regression lines show the obvious result: The more eerie a character appears, the less likable it is for both input devices. Note: We use the average values for eeriness and likability for the 3D models but used the not accumulated values for plotting the regression lines

A negative correlation between eeriness and likability can be observed with a factor of -0.707 for the HMD and -0.473 for the monitor with $p<$ 0.01 for both devices. The lines in Fig. 11 show, that the negative slope is increased for the HMD. This confirms that the measured extreme values for the HMD are more pronounced.

## Importance and implications for research and practice

This research on the one hand has revealed that there is a significant difference in the perception of humanlike characters between monitors and HMDs which are important for future design consideration in regards to the design of virtual content, especially virtual characters and on the other hand, it shows, that even if tried, it is only insufficiently possible to create an avatar in current SVR platforms that resembles oneself. For the UV study we can see in addition to what Kim et al. found [20], that HMDs elicit stronger negative emotions than other environments which is even more pronounced for characters that fall in the so-called UV. This implies a higher felt immersion in virtual environments accessed through an HMD instead of a monitor which is widely acknowledged already but brings us to further very important findings that need to be considered when either creating content for a specific output device such as HMD or a monitor or when porting content from a monitor application to an HMD application. Taking these results in consideration, when looking at the results of the second study where we measured the possibility to create an avatar that might resemble oneself, the findings of the UV study eventually needs to be added on top of this. Meaning, if you create an avatar using a custom avatar generator tool to use them within a SVR application, it could lead to even worse and unexpected results as the ones currently measured because it was measured on a 2D screen and not from within VR. Since some platforms also offer the possibility to upload a custom character, this leads to an imbalance within SVR applications. If, for example, someone is able to recreate himself using highly sophisticated 3D modeling tools or owns expensive hardware that could help him to do so, he would be able to control his/her look and feel way more compared to someone who does not. Also, none of the platforms’ customization tools offered the possibility to add inclusive parameters such as wheel chairs or prosthetics that could be added to the avatar. Luckily these problems are not unheard by the platform owners as well as researcher. We can see a large number of new publications that investigate and improve AI-based avatar creation from smartphone cameras or other standard RGB cameras. For example by the time this work was written, HTC released their new facial tracker camera to the public.^11^ With this technology, people are able to display their lower face movements (especially the are around the mouth) on their virtual avatar. Also, several researchers investigate how nonverbal cues can be brought into VR by using external cameras such as a Microsoft Azure Kinect or cameras mounted on the HMD.

## Limitations, conclusion and outlook

In this paper, we focused on the exploration of two objectives considering visual appearance of avatars. First, we took inventory of selected SVR platforms focusing visual self-representation and resemblance. Further, explored the “uncanny valley” effect regarding the visual appearance of avatars and compared rendering in HMDs with monitors.

We can conclude that there are important findings regarding static characters in virtual environments at this stage of research. Avatars are perceived comparatively differently via an HMD than on a monitor. In more advanced steps, it is certainly useful to include the influence of motion in the research, similar to what Mori proposed in his graph for moving entities (see Fig. 2). In addition to motion, so-called “mid-fidelity” interaction, as proposed by McMahan, could also lead to a deeper valley when combined with characters. We think that many current technologies that attempt to recreate a virtual human using camera data (RGB, depth) produce results that could tend to fall directly into the UV. For example, artifacts that make someone look just a little less human could be enough to fall into the UV. This could have a negative effect—unconsciously—on self-presentation in online meetings, which is reflected back into real life. In summary, HMDs are a technology where virtual characters in particular are perceived quite differently from a 2D display such as a monitor, including effects such as proximity and uncanniness.

Over all SVR platforms, we discovered a large variety in terms of character style, likability, eeriness and human likeness. One indication of our SVR platform inventory is that creating an avatar that resembles a real person with the current platform’s own editors is a difficult endeavor. The versatile SVR platform providers are apparently exploring different solutions to address this challenge. Influenced by the wants of the target groups, use cases, and the tracking methods included, the concepts can vary. While most platforms provide classical avatar creation tools, some allow free customization using 3rd party tools for extensive custom character creation. Others focus on the usage of scanning technologies to automatically create avatars or mix different approaches. Although our characters were almost lined up in a linear fashion in regards to the avg. human likeness, many of them are made in a rather cartoonish style than being realistic representation of a human being. This however may reduce the resemblance but also might purposely be done to avoid avatars to fall into the UV. Further, this can lead to finding a “sweet spot” where avatars have as much human likeness and resemblance to the depicted subject as possible without being perceived as eerie and fall into the UV.

While for platforms that focus on entertainment, human likeness, resemblance and eeriness might not play an important role or even is unwanted, this might not be true in business use cases where recognition of a potential interlocutor by its physical representation is essential. However, as long as there exist no adequate solution that is accepted by a wide audience, the advantages of SVR applications compared to conventional conferencing tools, such as a better transmission of proxemics or eye contact, are unlikely to be utilized.

## Footnotes

1 In 2016, they proposed their 10-year roadmap, which includes social VR as a separate item alongside artificial intelligence or drones: https://about.fb.com/news/2016/04/f8-2016-day-1/.

2 By the time this paper was written, many people had to work from home because of safety measurements that have been taken place in regards to the SARS-CoV-2 virus.

3 Image found in a german news - article by Eva Wolfangel in which she was documenting the differences in virtual meetings. https://www.riffreporter.de/vr-reporterin/schoene-neue-zoom-welt/ [last accessed: 2020-01-16].

4 By $\bar{x}$ we mean the average value over all measures of the regarding variable x.

5 Note that for the regression lines in the plot we calculated a simple linear regression.

6 While findings might be similar in regards to character presentation, we did not explicitly examine it and therefore are not using it.

7 See more: https://unity.com/.

8 The VG Resource https://www.vg-resource.com/.

9 See more: https://www.mixamo.com/  Product of Adobe$^{\mathrm{TM}}$ Corp.

10 This was also Mori’s approach in the original graphic of the UV.

11 https://www.vive.com/de/accessory/facial-tracker/
